# Editorial Notes

**Published:** 1883-05

**Authors:** 


### EDITORIAL NOTES.

A LETTER recently received from our colleague, Dr. Davidson, states that he is enjoying the hospitals of London, and appreciating the advantages there afforded for clinical observation and study. He expects to go over to Paris for a brief visit, and return to Buffalo about the middle of May.

AMONG the evidences of enterprise in the periodical medical literature of this country, the change of the *Maryland Medical Journal*, published in Baltimore, to a weekly publication, is especially to be noticed. This periodical has been one of our most valuable exchanges, and we congratulate the editors upon the increased support which warrants them to make the further venture of a weekly issue.

THE thirty-fourth annual session of the American Medical Association will be held in Cleveland, Ohio, on Tuesday, Wednesday, Thursday and Friday, June 5, 6, 7 and 8, 1883, commencing on Tuesday at 11 A. M. The proceedings of the body are always of great professional interest. The sessions call together the ablest men of this and other countries, and we have no doubt the coming meeting will be characterized by the same earnest effort in behalf of medical science as has been noticed in the past sessions.

WE have received the first number of the *American Psychological Journal*, issued by the National Association for the Protection of the Insane and Prevention of Insanity, a quarterly, edited by Joseph Parrish, M. D. of Burlington, N. J., and published by P. Blakeston, Son & Co., 1012 Walnut street, Philadelphia. Dr. Parrish is assisted by five associate editors. The subscription is $2.00 per year. The table of contents furnishes contributions from the ablest writers in this special department of medicine. We commend the journal to those of our readers who are interested in the study and treatment of insanity.

A special meeting of the Erie County Medical Society was held at the College Buildings, April 11th, to take action in regard to the bill introduced into the Legislature to establish a State Board of Medical Examiners. The following resolutions were unanimously adopted:

*Whereas*, The Committee on Legislation of the Medical Society of the State of New York did, at the last meeting of the society, report a proposed bill for the purpose of regulating the licensing of physicians, by the creation of a State Board of Examiners, which Board shall alone have power to grant licenses to practice medicine in this State; and

*Whereas*, The said State Medical Society did accept the report of the said Committee on Legislation, and did approve and recommend to the Legislature the passage of the said proposed bill; and

*Whereas*, The said bill is now before the said Legislature; therefore

*Resolved*, That the Medical Society of the County of Erie hereby approve of the bill, entitled an act to regulate the licensing of practitioners of physic and surgery, now before our State Senate.

*Resolved*, That it is the belief of this Society that the present system of training physicians does not command the entire confidence of the profession or of the people, and that the changes contemplated in the proposed law will be in the way of improvement.

An Instantaneous Light.—Such in a word is the unique apparatus on exhibition at the rooms of the Portable Electric Light Co., 22 Water street, Boston. It occupies the space of only five square inches and weighs but five pounds, and can be carried with ease. The light, or more properly lighter, requires no extra power, wires or connections, and is so constructed that any part can be replaced at small cost. The chemicals are placed in a glass retort; a carbon and zinc apparatus, with a spiral platinum attachment, is then adjusted so as to form a battery, and the light is ready. The pressure on a little knob produces an electric current by which the spiral or platinum is heated to incandescence. The Portable Electric Light Company was recently incorporated, with a capital of $100,000, under the laws of Massachusetts. The usefulness of the apparatus and the low price ($5) will no doubt result in its general adoption. Some of the prominent business men of the State are identified with this enterprise. In addition to its use as a lighter, the apparatus can also be used in connection with a burglar-alarm and galvanic battery.—*Boston Transcript, Dec.* 30.

